# Seroprevalence of Measles-, Mumps-, and Rubella-Specific Antibodies in Future Healthcare Workers in Serbia: A Cross-Sectional Study

**DOI:** 10.3390/vaccines13070700

**Published:** 2025-06-27

**Authors:** Ana Banko, Andja Cirkovic, Vladimir Petrovic, Mioljub Ristic, Vladimir Vukovic, Dobrila Stankovic-Djordjevic, Danijela Miljanovic

**Affiliations:** 1 Institute of Microbiology and Immunology, Faculty of Medicine, University of Belgrade, 11000 Belgrade, Serbia; danijela.karalic@med.bg.ac.rs; 2 Institute for Medical Statistics and Informatics, Faculty of Medicine, University of Belgrade, 11000 Belgrade, Serbia; andja.cirkovic@med.bg.ac.rs; 3 Institute of Public Health of Vojvodina, 21000 Novi Sad, Serbia; vladimir.petrovic@izjzv.org.rs (V.P.); mioljub.ristic@mf.uns.ac.rs (M.R.); vladimir.vukovic@mf.uns.ac.rs (V.V.); 4 Faculty of Medicine, University of Novi Sad, 21000 Novi Sad, Serbia; 5 Department of Microbiology and Immunology, Faculty of Medicine, University of Nis, 18000 Nis, Serbia; dobrila.stankovic.djordjevic@medfak.ni.ac.rs

**Keywords:** seroprevalence, MMR, measles, mumps, rubella, healthcare workers (HCWs), women of childbearing age

## Abstract

Background/Objectives: Measles, mumps, and rubella (MMR) continue to pose a significant public health challenge due to insufficient immunization coverage. This study aimed to provide the first seroprevalence data against MMR and to explore self-reported immunity among future healthcare workers (HCWs) in Serbia, including women of childbearing age. Methods: We included 1296 future health care workers (HCWs) aged 19 to 29, born in Serbia. All HCWs supplied a blood sample for serology and filled in a questionnaire. Antibodies were measured using an enzyme immunoassay against measles, mumps, and rubella (MMR). Results: Anti-measles, -mumps, and -rubella seronegativity rates were 25.6%, 26.5%, and 4.4%, respectively, among future HCWs in Serbia. The mumps seronegativity rate was significantly higher in the oldest (27–29-year) age group, accompanied by significantly lower anti-mumps IgG GMCs in the same age group compared to younger participants (*p* = 0.035 and *p* < 0.001, respectively). Anti-mumps seronegativity also increased significantly across birth cohorts, from the youngest to the oldest (*p* = 0.004). Furthermore, anti-mumps IgG antibody GMCs were significantly higher among females, those who attended nursery/kindergarten, and unvaccinated individuals (*p* = 0.050, *p* = 0.020, and *p* = 0.005, respectively). Finally, older age and unvaccinated status were identified as independent factors associated with anti-measles and anti-mumps seronegativity among future HCWs in Serbia. Conclusions: The cross-sectional seroprevalence data revealed insufficient seroprotection in this population of particular importance, i.e., future HCWs, and women of childbearing age. These results strongly support the national recommendations for the mandatory vaccination of these populations. Identified immunity gaps should be closed promptly by strategic, targeted serologic screening, followed by vaccination of those lacking MMR antibodies.

## 1. Introduction

Despite the availability of safe and effective vaccines, measles, mumps, and rubella continue to pose a significant public health challenge due to insufficient immunization coverage. In 2023, an estimated 107,500 individuals died from measles globally, primarily children under the age of 5 years [1]. In the European region, the World Health Organization (WHO) targeted the elimination of measles, rubella, and, in particular, congenital rubella syndrome (CRS) [2]. However, the latest report of the Strategic Advisory Group of Experts (SAGE) on Immunization, published in March 2024, revealed that since 2019, the numbers of unvaccinated and under-vaccinated children has increased in both high-income and middle-income countries, reaching almost 1.5 million [1]. Notably, measles cases increased 62-fold in 2023, compared to 2022. Of these, 95% were reported from middle-income countries. Furthermore, for the last twelve months (from 1 March 2024, to 28 February 2025), data on measles cases in the European Union and European Economic Area (EU/EEA) show a considerable rise in notifications compared to 2023. In line with the seasonal pattern of measles, a further increase is expected during the spring of the current year [3]. According to the 2022 annual report published by the National Health Institute of the Republic of Serbia, the epidemiological situation regarding measles was deemed to be threatening [4]. Between 1 January 2024, and 30 November 2024, in Serbia, 694 measles cases were registered [5].

The history of measles, mumps, and rubella mandatory vaccination in Serbia includes three key dates, i.e., 1971, 1981, and 1993. Firstly, measles vaccination became mandatory in 1971, followed by the implementation of the measles-mumps (MM) vaccine in 1981. Immunization against rubella became part of the national immunization program in 1993, after the introduction of the measles, mumps, rubella (MMR) single-dose vaccine. In 1996, the two-dose schedule of the MMR vaccine was established, initially with the second dose administered at the age of 12 before this was switched to the age of 7 in 2006 [6]. Despite long-standing prevention programs and systematic vaccination against communicable diseases, several barriers have been identified to optimal immunization over the last few decades in Serbia: vaccine hesitancy, limited vaccine availability, and occasional interruptions in vaccine supply [7,8]. Even over the two decades preceding the COVID-19 pandemic’s impact on immunization hesitancy, an analysis in Serbia showed a significant decline in coverage through multiple primary vaccinations and revaccination, including MMR [9]. In the last measles epidemic in Serbia, between 2017 and 2018, approximately 5800 cases were registered, of which 93% involved unvaccinated, incompletely vaccinated, or without known vaccination status individuals. A third of the patients required hospitalization due to the severity of the clinical course and complications; 15 infected people died, of which four were children under the age of five. Fifty cases of measles from the previous year, 2023, were also mostly unvaccinated (94%) [10].

To implement the recommendations of the World Health Organization (WHO), the SAGE, and the European Centre for Disease Prevention and Control (ECDC) related to closing immunity gaps by establishing catch-up vaccination activities and determining country-specific immunization strategies, it is necessary to assess the missing serosurvey data. To date, there is no available evidence of measles, mumps, and rubella seroprevalence in Serbia, except for subnational data from the Autonomous Province (AP) of Vojvodina [6,11]. Moreover, our country is still missing a national vaccination registry. Therefore, this study aimed to provide the first seroprevalence data on IgG antibodies against measles, mumps, and rubella and to explore self-reported immunity among future healthcare workers (HCWs) in Serbia, including women of childbearing age. Thus, identifying seronegative individuals may help predict or even prevent potential outbreaks in the future.

## 2. Materials and Methods

### 2.1. Study Design and Data Collection

This cross-sectional study enrolled 1296 apparently healthy future HCWs in Serbia. From September 2023 to August 2024, healthcare students attending the faculties of medical sciences at the three largest universities (University of Belgrade, Novi Sad, and Niš) were invited to participate in the project. In addition to studying medical sciences, only students born in the territory of the Republic of Serbia, due to the implementation of the same mandatory immunization schedule for all research participants aged 19 to 29 as the target population, were eligible to participate in the research. Each student voluntarily filled in a semi-structured questionnaire and provided a blood sample for serological analysis. The questionnaire was divided into three groups of questions: Group I referred to personnel data (year of birth, university and study year attending, and gender), Group II referred to socio-epidemiological data (place of residence, migration, nursery/kindergarten attendance), and Group III referred to self-reported vaccination status (with an unknown number of doses) according to the personal vaccination record (if they had it), consultation with parents, or just by memory. Migration was defined as relocation from one settlement to another, typically occurring by the end of primary education. This period was chosen because mandatory vaccination ends at that age [12]. Between 1995 and 2005, mandatory immunization in Serbia was conducted against eight communicable diseases, i.e., tuberculosis, diphtheria, tetanus, pertussis, poliomyelitis, mumps, measles, and rubella, while vaccines against hepB and diseases caused by Hib were introduced in the national mandatory immunization schedule in 2005 and 2006, respectively [13]. Based on their place of birth, our participants were classified into two groups: major urban and other urban and rural settlements. Major urban areas included the largest urban settlements (Belgrade, Novi Sad, Kragujevac, and Niš), while other areas included other urban and rural settlements in Serbia. Personal and confidential information was removed, except for the information previously mentioned in the three groups of questions. Data regarding immunization coverage in Serbia were obtained by reviewing the data of the Institute of Public Health of Serbia “Dr Milan Jovanovic Batut”.

### 2.2. Serological Testing—MMR IgG ELISA

One blood sample (5–10 mL) was collected from each enrolled student in a blood tube with a clot activator. Sera were separated after centrifugation, placed into sterile tubes, and transported to the Virology Laboratory of the Institute for Microbiology and Immunology, Faculty of Medicine, University of Belgrade, where they were stored at −20 °C until further analysis. After each round, in which 90 residual serum samples were collected, samples were analyzed, ensuring that the period between sampling and testing did not exceed 3 weeks for each sample.

Anti-measles, anti-mumps, and anti-rubella IgG were identified and measured using commercial ELISAs according to the manufacturer’s instructions in collected sera (Euroimmun, Lubeck, Germany). An automated processor (Analyzer I-4P, Euroimmun) was used for the analysis. Standard calibrators were used in each assay to calculate index values and optical density (OD) ratios, which serve as quantitative measures of IgG antibody titers. Absorbance was measured using a Multiscan FC microplate reader (Thermo Scientific, Waltham, MA, USA) at a wavelength of 405 nm with background subtraction at 650 nm. All assays met the predetermined quality control measures, as determined by positive, negative, and blank controls. According to the manufacturer’s instructions, the results were interpreted as follows. For measles, negative: <200 IU/L; equivocal: ≥200 and <275 IU/L; positive: ≥275 IU/L. For mumps, negative: <16 RU/mL; equivocal: ≥16 and <22 RU/mL; positive: ≥22 RU/mL. Finally, for rubella, negative: <8 IU/mL; equivocal: ≥8 and <11 IU/mL; positive: ≥11 IU/mL. The lower detection limit of anti-measles IgG was 8 IU/L, and the upper detection limit was 5000 IU/L, with a linearity range of 52–4865 IU/L. Antibody levels above the quantification range (≥5000 IU/L) were set to 5000 IU/L. The test shows a specificity and a sensitivity of 100% each. The anti-mumps IgG linearity range was 4–164 RU/mL, with a lower detection limit of 0.3 RU/mL and an upper detection limit of 200 RU/mL (antibody levels above the quantification range, i.e., ≥200 RU/mL, were set to 200 RU/mL), and the sensitivity amounted to 99.3%, with a specificity of 100%. The lower detection limit of anti-rubella IgG was 0.3 IU/mL, and the upper detection limit was 200 IU/mL, with a linearity range of 5–183 IU/mL. Antibody levels above the quantification range (≥200 IU/mL) were set to 200 RU/mL. The specificity was 100%, with a sensitivity of 99.6%.

### 2.3. Sample Size

This research is part of a larger scientific project examining the seroprevalence of various viruses that may impact reproductive health. Therefore, the calculation of the project sample size considered limited seroprevalence data in Serbia or data from the most closely related European population, since data for our country did not exist. The sample size was calculated based on a minimum estimated seroprevalence of 12% (the seroprevalence of Herpes Simplex Virus type 2 in Serbia), a precision of 0.02, and a 95% level of significance. The estimated sample size of 1014 covered all project aims, including the seroprevalence of measles, mumps, and rubella. Considering the number of students attending the three universities involved in this study, with Belgrade University being the largest among them, the estimated share of all enrolled study respondents was expected to be 50% from the University of Belgrade, 25% from the University of Niš, and 25% from the University of Novi Sad to make a representative sample of future HCWs of childbearing age as the target population for the analysis. The number of students attending the faculties of medical sciences at the three included universities in Serbia during school year 2023/24 and their participation rates were 12.1% (739 out of 6092), 9.4% (383 out of 4074), and 5.3% (174 out of 3290) for University of Belgrade, Novi Sad, and Niš, respectively.

### 2.4. Statistical Analysis

Categorical data are presented as absolute and relative numbers in percentages. Numerical data are described by the arithmetic mean with standard deviation. Normality was assessed using mathematical (Shapiro-Wilk test, Kolmogorov-Smirnov test, skewness, kurtosis, coefficient of variation) and graphical (histogram, box plot) methods. For anti-measles, anti-mumps, and anti-rubella IgG antibody titers, Geometric Mean Concentration (GMC) was used. After logarithmic transformation, the arithmetic mean and standard deviation were returned to their original values by inverse transformation. To test the difference in the distribution of categories of nominal data across independent samples, a chi-square test was applied. One-way ANOVA with Tuckey post hoc testing was applied to test the difference in arithmetic means of numerical data with normal distribution between independent groups. To evaluate factors associated with measles, mumps, and rubella seronegativity, where the outcome was defined as dichotomous, univariate and multivariate binary logistic regression analyses were performed. All evaluated characteristics in the study population (gender, age groups, region of origin, nursery/kindergarten attendance, migration, and self/reported vaccination status) were evaluated in our univariate analysis. Afterward, factors that were significant in the univariate analysis at the 0.05 level were included in the multivariable model. Also, all potential confounding factors (age, gender, migration, region of origin, attending nursery/kindergarten) were considered in the multivariable modeling. Odds ratio (OR), 95% confidence interval of odds ratio (95% CI OR), and *p*-value are reported for the regression models. Violin plots were created using an interactive online dot plot [14]. Statistical significance was determined at a *p*-value threshold of ≤0.05. The analysis was conducted using the statistical software released by IBM Corp. in 2023 (IBM SPSS Statistics for Windows, Version 29.0.2.0 Armonk, NY, USA).

## 3. Results

This cross-sectional study of the seroprevalence of measles, mumps, and rubella IgG antibodies in the population of future HCWs in Serbia comprised a total of 1296 respondents. All participants were born between 1995 and 2005, with an average age of 22.9 ± 2.2 years, and a female-to-male ratio of 4:1.

### 3.1. Seroprevalence of Measles, Mumps, and Rubella IgG Antibodies in Serbian Future HCWs

The seroprevalence of MMR IgG antibodies in Serbian future HCWs aged 19–29 years is shown in Table 1. Anti-measles IgG was detected in 63.0% (95% CI, 60.3–65.7) of participants, while 11.3% (95% CI, 9.7–13.2) showed equivocal results, and 25.6% (95% CI, 23.3–28.1) had negative results. Anti-mumps IgG was detected in 60.2% (95% CI, 57.5–62.9) of participants, while 13.3% (95% CI, 11.5–15.3) showed equivocal results, and 26.5% (95% CI, 24.1–29.0) had negative results. Anti-rubella IgG was detected in 92.2% (95% CI, 90.6–93.6) of participants, while 3.4% (95% CI, 2.5–4.5) showed equivocal results, and 4.4% (95% CI, 3.4–5.7) had negative results.

Table 1 also presents the seroprevalence of IgG antibodies against measles, mumps, and rubella in future HCWs according to the evaluated population characteristics. The seropositivity rate for mumps was significantly higher among participants aged 19–20 years compared to other age groups (19–20 years 68%, 21–23 years 60%, 24–26 years 61%, 27–29 years 48%; *p* = 0.035). Additionally, the prevalence of positive anti-measles and anti-mumps IgG antibodies was significantly higher in individuals who reported being vaccinated than in those who reported being unvaccinated (anti-measles: 64% vs. 56%, *p* = 0.025 and anti-mumps: 62% vs. 49%, *p* = 0.017, respectively). There was no statistically significant difference in anti-measles, -mumps, and -rubella IgG antibodies by gender (*p* = 0.315, *p* = 0.162, and 0.248, respectively), region of origin (*p* = 0.785, *p* = 0.894, and *p* = 0.234, respectively), nursery/kindergarten attendance (*p* = 0.962, *p* = 0.072, and *p* = 0.124, respectively), or migration (*p* = 0.944, *p* = 0.816, and *p* = 0.282).

### 3.2. Anti-Measles, Anti-Mumps, and Anti-Rubella IgG Antibody Titers in Serbian Future Healthcare Workers

**Figure 1:**
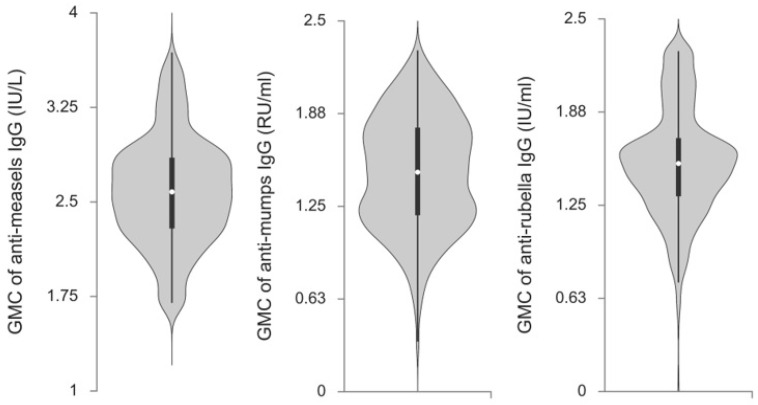
Violin plot of GMCs of anti-measles, anti-mumps, and anti-rubella IgG antibodies in future HCWs in Serbia. The width of a violin plot corresponds to the frequency of specific value. Each violin plot contains a box plot inside it, where a white dot represents the median, the end of a black bold line represents the interquartile range, and whiskers represent minimum and maximum values.

The titer of anti-measles, anti-mumps, and anti-rubella IgG antibodies in future HCWs aged between 19–29 years expressed as GMCs are presented in Figure 1. The average value of anti-measles IgG antibody titers was 384.59 ± 2.65 IU/L, of anti-mumps IgG antibody titers was 29.92 ± 2.40 RU/mL, and of anti-rubella IgG antibody titers was 34.04 ± 2.34 IU/mL.

GMCs were evaluated based on gender, age groups, region of origin, nursery/kindergarten attendance, migration status, and self-reported vaccination status (Table 2). First, it was determined that anti-measles GMCs of IgG antibodies were significantly higher in individuals who reported being vaccinated than in those who reported being unvaccinated (*p* = 0.003), while they did not differ according to gender (*p* = 0.225), age groups (*p* = 0.056), region of origin (*p* = 0.989), nursery/kindergarten attendance (*p* = 0.210), and migration (*p* = 0.621). Anti-mumps GMCs of IgG antibodies were significantly higher in females than in males (*p* = 0.050), in individuals who had not attended nursery/kindergarten than in those who had (*p* = 0.020), and among individuals who reported being vaccinated than in those who reported being unvaccinated (*p* = 0.005), but it remained the same among individuals from major urban settlements and those from rural settlements (*p* = 0.755). Also, anti-mumps GMCs of IgG antibodies were significantly different between age groups. Thus, they were the highest among the youngest individuals (*p* < 0.001). Between-group comparisons revealed that individuals aged 19–20 years had significantly higher anti-mumps GMCs than those aged 21–23, 24–26, and 27–29 years (*p* = 0.002, *p* = 0.002, and *p* < 0.001, respectively). Finally, there was no difference in anti-rubella the GMCs of IgG antibodies according to any of the evaluated characteristics (*p* = 0.987, *p* = 0.067, *p* = 0.743, *p* = 0.144, *p* = 0.881, *p* = 0.771, respectively).

**Figure 2:**
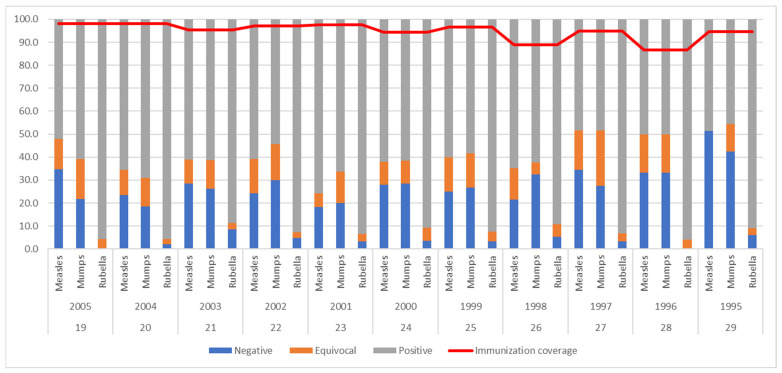
Seroprevalence of measles, mumps, and rubella IgG antibodies and MMR vaccine immunization coverage (2nd dose) in future Serbian HCWs by year of birth from 1995–2005 (n = 1296). Vaccination against MMR explanation: The first dose of the MMR vaccine was administered at 12–15 months for all birth years. For respondents born between 1995 and 1999, the second dose of the MMR vaccine was administered at the age of 12, whereas it was administered at the age of 7 for those born between 2000 and 2005. Thus, there was a catch-up period from 2006 to 2011, because the second dose was given at the age of 12 to children born between 1995 and 1999 and at the age of 7 to those born between 2000 and 2005.

Seroprevalence, depending on the year of birth, was evaluated and is presented in Figure 2 along with immunization coverage per year. It was determined that there was a significant increase in the trend of anti-mumps seronegativity from the youngest to the oldest respondents (*p* = 0.004), whereas there was no significant change in seroprevalence depending on the birth year for anti-measles and anti-rubella IgG antibodies (*p* = 0.145 and *p* = 0.784). During the same period, immunization coverage ranged from the minimum of 88.8% in 1998 to the maximum of 98.1% in 2004.

### 3.3. Factors Associated with Measles, Mumps, and Rubella Seronegativity in Future Serbian HCWs

We analyzed factors associated with seronegativity for measles, mumps, and rubella in future HCWs in Serbia; the results of the univariate and multivariate logistic regression analyses are presented in Table 3. Using the 21–23 years age group as a comparator (i.e., that with the lowest measles seronegativity), the prevalence of measles seronegativity was significantly higher in the 27–29 years age group (OR = 2.151; 95% CI 1.34–3.45, *p* = 0.001). Self-reported non-vaccination status was also significantly associated with measles seronegativity (OR = 1.730; 95% CI 1.18–2.54, *p* = 0.005). After adjustment for gender, region of origin, attending nursery/kindergarten, and migration as a potential confounders, both factors remained associated with measles seronegativity. Using the 19–20 years age group as a comparator (i.e., that with the lowest mumps seronegativity), and after adjusting for potential confounders, the prevalence of mumps seronegativity was significantly higher in the 24–26 and 27–29 years age groups (OR = 1.796; 95% CI 1.13–2.85, *p* = 0.013 and OR = 2.217, 95% CI 1.21–4.05, *p* = 0.010, respectively). Self-reported non-vaccination status was also significantly associated with mumps seronegativity (OR = 0.597; 95% CI 0.41–0.88, *p* = 0.009). There were no factors associated with rubella seronegativity.

### 3.4. Combined Seroprevalence Against Measles and Rubella or Measles, Mumps, and Rubella as a Proxy for the Vaccination Status

To determine the actual proportion of seronegative respondents (unvaccinated individuals who had not suffered from measles, mumps, and rubella) in our study cohort, we analyzed the combined serostatus of IgG antibodies against measles, mumps, and rubella. Double-negative (anti-measles and anti-rubella IgG antibody ELISA negative) and triple-negative (anti-measles, anti-mumps, and anti-rubella IgG antibody ELISA negative) results were present in the cohort born between 1995 and 2005, i.e., 2.9% was double negative and 2.4% was triple negative, without significant difference between those born 1995–1999 and 2000–2005, *p* = 0.603 and *p* = 0.435, respectively (The second dose of the MMR vaccine was administered at the age of 12 from its introduction in 1994 while, since 2000, the second dose has been administered at the age of 7; see Supplementary Table S1). Additionally, there was no significant difference in double and triple negativity between age groups (19–20, 21–23, 24–26, 27–29) (*p* = 0.447, and *p* = 0.395, respectively); see Supplementary Table S1. If we take the rubella seronegative result (*n* = 58) as the reference status (since it has previously been shown that high seropositivity is maintained from generation to generation without imbalance or decline), it was found that 45 individuals were also negative for mumps and 38 for measles (77.6% and 65.5% of 58 rubella negative), which represents 3.5% and 2.9% of the total number of participants in the study, respectively. If rubella seronegative and equivocal results (*n* = 102) are taken as the reference, the number of individuals who were also negative for mumps was 72 (70.6%), and for measles 63 (61.8%), representing 5.5% and 4.9% of the total number of individuals, respectively (see Supplementary Table S2). During the same period (1995–2005, 1995–1999, and 2000–2005), a significant moderate positive correlation was observed in anti-measles, anti-mumps, and anti-rubella IgG antibodies (see Supplementary Table S3).

## 4. Discussion

The measles outbreaks in 2023 and 2024, especially in Europe, raised concerns about underestimated existing immunity gaps, a decrease in vaccination coverage due to the recent pandemic, and/or the waning of protective antibody levels in vaccinated individuals over time [15]. The results of this study provided the first MMR seroprevalence data for the Serbian young adult population among future HCWs. With even a quarter of the measles and mumps seronegative individuals investigated, we identified a potentially vulnerable group. In the absence of a national immunization registry, but with a demonstrated association between self-reported non-vaccination status and measles or mumps seronegativity, the obtained data may help evaluate seroprotection and develop strategic measures to prevent potential outbreaks in the future.

The target group in this research provided important insights into the specific risks of future HCWs and women of reproductive age. It is known that HCWs are particularly susceptible to transmissible diseases and may play a role in the nosocomial transmission of infectious diseases, posing a risk to themselves and their patients, especially during outbreaks. Previously reported nosocomial epidemics of measles or mumps involved both patients and HCWs [16,17,18,19]. European seronegativity rates in HCWs for measles range between 1.6% and 19%, for mumps between 10.8% and 31.2%, and for rubella between 2.4% and 7.7% [20,21,22,23]; these values did not align with the specific characteristics exhibited in young HCWs, as shown in our study. It turns out that the seroprotection of young HCWs was often insufficient, estimated at 66–94% for measles, 53–82% for mumps, and 76–88% for rubella [22,23,24]. Notable was the rarity of consistent values for all three viruses across the same studies, except in pediatric HCWs from Denmark [21]. Compared to other studies, our respondents showed lower seropositivity for measles (63%), values in the lower range for mumps (60.2%), and surprisingly higher seropositivity for rubella (92.2%). Considering the differences in national immunization programs, and particularly in the timing of receiving the first and second doses of the MMR vaccine, it may be challenging to compare the seroprevalence values of different countries. On the other hand, what is consistently emphasized in all studies is the need for special caution for this unambiguously vulnerable group.

To estimate whether a lack of antibodies might reflect vaccination status, we matched the proportion of seronegative individuals for more than one type of antibody, estimated to be 5.5% of unvaccinated individuals [15]. The combined obtained seronegativities did not differ between the group that received the second dose of the MMR vaccine at the age of 7 and the group that was revaccinated at the age of 12.

Mumps had the highest seronegativity rate of the MMR vaccine components [21,25,26]. In the vaccinated population, three key phenomena could clarify this seronegativity: primary vaccine failure or an individual deficiency in seroconversion after vaccination, low antibody avidity indexes (AI), and waning immunity. Thus, seroconversion for mumps is estimated to be 93.3%, while higher seroconversion rates are estimated for measles (96%) and rubella (98.3%) [24]. The effectiveness of the MMR vaccine was also the lowest in preventing mumps (86%) compared to measles (96%) and rubella (89%) [27]. Despite mumps having a low AI, measles and rubella are associated with high AI [28]. As a demonstration of the poor induction of lasting high-concentration antibodies, mumps antibodies decrease in both the mean antibody titer (65%) and the mean AI (24%) in the 20-year follow-up. Meanwhile, the mean AI for measles decreases by 8%, while it remains unchanged for rubella [28]. The estimated protection against all three components of the MMR vaccine is therefore 10–15 years [21,29,30]. This gradual loss of immunity could explain the age-dependent decline in seroprevalence of anti-mumps IgG antibodies by more than 30% in our oldest age group, as well as the highest anti-mumps GMCs of antibodies among the youngest individuals, as reported in a German serosurvey [25]. Moreover, our study reported insufficient MMR coverage among the oldest participants.

The obtained equivocal results were related to 11.3% of respondents for measles, 13.3% for mumps, and 3.4% for rubella antibodies, similar to reports by other authors, which showed that the equivocal frequency was higher in individuals younger than 29 years [25,31]. However, the immunological implications of low or missing antibody titers in vaccinated individuals remain insufficiently defined [32,33]. Some publications have demonstrated sufficient anti-measles antibody neutralizing capacity in vaccinated persons even when titers were equivocal or negative [34]. Despite these laboratory experiments, real-world data yield different outcomes, such as hospital outbreaks of measles and mumps involving previously vaccinated HCWs [16,32,35]. Outbreaks of measles mainly occur in susceptible individuals, i.e., those who are not infected, not vaccinated, or too young to be vaccinated, or due to primary vaccine failure [36,37]. The same susceptibility was reported in previous Serbian outbreaks [10,11,38]. However, a particular problem arises if population immunity remains below the level required for herd immunity. In such circumstances, breakthrough infections are inevitable [39]. Furthermore, the waning immunity of maternal antibodies may lead to a gap in protection for children in their first year of life and pose a major risk for disease development in newborns, which is commonly associated with severe disease, the development of complications, and late sequela, i.e., subacute sclerosing panencephalitis [40]. During the measles outbreaks in 2023 in Mumbai, India, 31% of the measles cases reported were infants below 9 months of age [41]. The estimated duration of maternal vaccination protection against measles is only 3.3 months after birth [42]. The consequences of rubella infection during pregnancy are also of particular importance, because it might lead to spontaneous abortion or preterm birth. CRS manifests as cataracts, heart defects, hearing impairment, and mental retardation [43,44]. Therefore, women of childbearing age with unknown immune status should be offered serology testing and/or the MMR vaccine before potential pregnancy, as part of routine prevention [25,45,46].

Due to the previously described critical limitations in vaccine efficacy and effectiveness, but also because immunization coverage is declining and vaccine hesitancy is becoming more commonplace, revising previous global and national immunization policies has already been suggested by many European countries [8,20,21,47]. Those that are related to HCW vaccination policies include the following: routinely checking HCWs’ serostatus; in cases where there is no evidence of protection, HCWs should be offered vaccination; vaccinations should be recorded. Although the laws of the Republic of Serbia impose mandatory MMR immunization and particularly rules for seronegative HCWs, women who are planning pregnancy, and other defined vulnerable individuals [48,49], the fact is that serological tests are not strategically and routinely performed in immune status screenings to identify those who would benefit from vaccination [12]. This is particularly critical, given the lack of a national vaccination registry.

In the absence of such a registry, it is challenging to identify and address other anomalies in immunization calendar compliance, such as delayed first MMR doses, low second-dose coverage rates, and vaccine hesitancy. Moreover, it is complex to measure the direct impact of interruptions in vaccine supplies and their limited availability, leading to occasional disruptions of immunization activities, such as those during 2012–2015 [9,11]. By analyzing the data for MMR vaccine coverage in the study cohort, we estimated variations ranging from 88.8% to 98.1%. Due to those periodic declines in immunization coverage, which continue in the generations born after the studied cohort, the risk of outbreaks has been repeated, including the risk to women of childbearing age [6]. It should also be noted that the MMR vaccine is a mandatory measure that is widely accessible, with coverage being very similar everywhere in Serbia [38]. Our study results support this, showing no differences in seroprevalences related to migration or the level of urbanity of participants’ place of birth. Finally, two more questions could be posed: what are the specific and individual impacts of the relatively recent outbreaks in 2014–2015 and 2017–2018, and to what extent could they influence the obtained serological markers, especially in participants from populated areas [38]?

### Strengths and Limitations of the Study

The strength of our study is reflected in the revelation of previously unknown primary national cross-sectional serosurvey data, as well as the large sample size of two populations at high risk of disease: future healthcare workers and women of reproductive age. The lack of a national vaccination register made it impossible to correlate the obtained serology and epidemiology results with the exact individual data concerning actual vaccination status and possible deviations concerning the national immunization calendar. Information on vaccinations or previous infections and illnesses, as in other studies, was incomplete and prone to error due to self-reporting through an epidemiological questionnaire [21,25,50]. The costs of the study included the ELISA method for serology testing, as a high-quality assay. However, additional costly methods like plaque reduction neutralization tests could be useful for retesting samples with equivocal ELISA results, as the relationship between measles or rubella antibody levels measured by ELISA does not always correlate with the degree of protection [15,25]. These results cannot be entirely extrapolated to the general population due to age limitations, the higher proportion of females, and the voluntary nature of participation in the study. Finally, we lack previous national serosurveys to evaluate or monitor changes in seroprevalence.

## 5. Conclusions

Our cross-sectional seroprevalence data revealed insufficient seroprotection in two populations of particular importance, i.e., future HCWs and women of childbearing age. The results of this study strongly support the recommendations of current national immunization regulations, particularly the mandatory vaccination of these populations. As eliminating or eradicating measles becomes increasingly complex, identified immunity gaps should be closed promptly by strategically introducing serologic screening for HCWs and women of childbearing age without documented evidence of vaccination or disease history, followed by more rigorously controlled immunization of those lacking MMR antibodies.

## Figures and Tables

**Table 1:** Seroprevalence of measles, mumps, and rubella IgG antibodies in Serbian future HCWs by gender, age groups, region of origin, nursery/kindergarten attendance, migration, and self/reported vaccination status.

Characteristics	Number of Samples	Measles			*p* *	Mumps			*p* *	Rubella			*p* *
		Negative % (95% CI)	Equivocal % (95% CI)	Positive % (95% CI)		Negative % (95% CI)	Equivocal % (95% CI)	Positive % (95% CI)		Negative % (95% CI)	Equivocal % (95% CI)	Positive % (95% CI)	
Total	1296	25.6 (23.3–28.1)	11.3 (9.7–13.2)	63.0 (60.3–65.7)		26.5 (24.1–29.0)	13.3 (11.5–15.3)	60.2 (57.5–62.9)		4.4 (3.4–5.7)	3.4 (2.5–4.5)	92.2 (90.6–93.6)	
Gender	Valid Number												
Male	264	29.2 (23.8–35.1)	9.8 (6.5–14.1)	61.0 (54.8–66.9)	0.315	31.1 (25.5–37.0)	13.3 (9.4–18.0)	55.7 (49.5–61.8)	0.162	3.8 (1.8–6.9)	1.9 (0.6–4.4)	94.3 (90.8–96.8)	0.248
Female	1026	24.9 (22.2–27.6)	11.6 (9.7–13.7)	63.5 (60.5–66.5)		25.4 (22.7–28.1)	13.2 (11.2–15.4)	61.5 (58.4–64.5)		4.6 (3.4–6.1)	3.8 (2.7–5.2)	91.6 (89.7–93.2)	
Age groups	Valid Number												
19–20	159	25.2 (18.6–32.6)	11.3 (6.8–17.3)	63.5 (55.5–71.0)	0.065	18.9 (13.1–25.8)	13.2 (8.4–19.5)	67.9 (60.1–75.1)	0.035	1.9 (0.4–5.4)	2.5 (0.7–6.3)	95.6 (91.1–98.2)	0.182
21–23	681	24.1 (20.9–27.5)	11.0 (8.8–13.6)	64.9 (61.2–68.5)		26.1 (22.9–29.6)	14.1 (11.6–16.9)	59.8 (56.0–63.5)		5.6 (0.4–7.6)	2.8 (1.7–4.3)	91.6 (89.3–93.6)	
24–26	355	25.6 (21.2–30.5)	12.4 (9.2–16.3)	62.0 (56.7–67.0)		28.7 (24.1–33.7)	10.7 (7.7–14.4)	60.6 (55.3–65.7)		3.9 (2.2–6.5)	5.1 (0.3–7.9)	91.0 (87.5–93.8)	
27–29	86	40.7 (30.2–51.8)	10.5 (4.9–18.9)	48.8 (37.9–59.9)		34.9 (24.9–45.9)	17.4 (10.1–27.1)	47.7 (36.8–58.7)		3.5 (0.7–9.9)	3.5 (0.7–9.9)	93.0 (85.4–97.4)	
Region of origin	Valid Number												
Major urban	632	25.0 (21.7–28.6)	11.9 (9.4–14.6)	63.1 (59.2–66.9)	0.785	26.7 (23.3–30.4)	12.8 (10.3–15.7)	60.4 (56.6–64.3)	0.894	5.2 (3.6–7.3)	4.0 (2.6–5.8)	90.8 (88.3–93.0)	0.234
Other urban and rural	664	26.2 (22.9–29.7)	10.8 (8.6–13.5)	63.0 (59.2–66.6)		26.4 (23.0–29.9)	13.7 (11.2–16.6)	59.9 (56.1–63.7)		3.8 (2.5–5.5)	2.9 (1.7–4.4)	93.4 (91.2–95.1)	
Attended nursery/kindergarten	Valid Number												
Yes	1140	25.7 (23.2–28.3)	11.4 (9.6–13.4)	62.9 (60.0–65.7)	0.962	27.3 (24.7–30.0)	13.6 (11.7–15.7)	59.1 (56.2–62.0)	0.072	4.7 (3.6–6.1)	3.2 (2.2–4.3)	92.1 (90.4–93.6)	0.124
No	153	24.8 (18.2–32.5)	11.1 (6.6–17.2)	64.1 (55.9–71.6)		20.3 (14.2–27.5)	11.1 (6.6–17.2)	68.6 (60.6–75.9)		2.0 (0.4–5.6)	5.2 (2.3–10.0)	92.8 (87.5–96.4)	
Migration	Valid Number												
Yes	551	25.4 (21.8–29.3)	11.1 (8.6–14.0)	63.5 (59.3–67.5)	0.944	26.0 (22.4–29.9)	14.0 (11.2–17.2)	60.0 (55.8–64.1)	0.816	3.4 (2.1–5.3)	3.6 (2.2–5.6)	92.9 (90.5–94.9)	0.282
No	741	25.6 (22.5–28.9)	11.6 (9.4–14.1)	62.8 (59.2–66.2)		27.0 (23.8–30.3)	12.8 (10.5–15.4)	60.2 (56.6–63.7)		5.1 (3.7–7.0)	3.2 (2.1–4.8)	91.6 (89.4–93.5)	
Self-reported vaccination status	Valid Number												
Yes	1138	24.1 (21.6–26.7)	11.7 (9.9–13.7)	64.2 (61.4–67.0)	0.025	25.5 (23.0–28.1)	12.9 (11.0–15.0)	61.6 (58.7–64.4)	0.017	4.1 (3.1–5.5)	3.4 (2.5–4.7)	92.4 (90.7–93.9)	0.513
No	152	34.8 (26.8–43.5)	9.6 (5.2–15.9)	55.6 (46.8–64.1)		34.8 (26.8–43.5)	16.3 (10.5–23.6)	48.9 (40.2–57.6)		5.9 (2.6–11.3)	3.4 (0.5–6.4)	91.9 (85.9–95.9)	

* for the level of significance of 0.05 according to chi-square test, Valid Number was reported for each of the variables. There were 6 missing data for gender, 15 for age, 3 for nursery/kindergarten attendance, 4 for migration, and 6 for self-reported vaccination status.

**Table 2:** Geometric mean concentration of anti-measles, anti-mumps, and anti-rubella IgG antibodies in future HCWs in Serbia by gender, age groups, region of origin, nursery/kindergarten attendance, migration, and self/reported vaccination status.

Characteristics	GMCs of IgG Antibodies					
	Anti-Measles (IU/L)	*p* *	Anti-Mumps (RU/mL)	*p* *	Anti-Rubella (IU/mL)	*p* *
Gender						
Male	359.75 ± 2.60	0.225	27.23 ± 2.43	0.050	34.04 ± 2.29	0.987
Female	389.94 ± 2.67		30.69 ± 2.39		34.04 ± 2.35	
Age groups						
19–20	427.56 ± 2.79	0.056	38.90 ± 2.39	<0.001	38.37 ± 2.26	0.067
21–23	384.59 ± 2.55		29.51 ± 2.33		33.11 ± 2.36	
24–26	384.59 ± 2.73		29.04 ± 2.48		34.51 ± 2.34	
27–29	299.23 ± 2.96		23.99 ± 2.43		28.84 ± 2.11	
Region of origin						
Major urban	384.59 ± 2.70	0.989	30.20 ± 2.44	0.755	34.36 ± 2.40	0.743
Other urban and rural	384.59 ± 2.62		29.72 ± 2.36		33.81 ± 2.28	
Attended nursery/kindergarten						
Yes	380.19 ± 2.67	0.210	29.38 ± 2.40	0.020	33.57 ± 2.33	0.144
No	422.67 ± 2.59		34.99 ± 2.38		37.41 ± 2.36	
Migration						
Yes	390.84 ± 2.59	0.621	28.97 ± 2.39	0.276	34.20 ± 2.34	0.881
No	380.19 ± 2.70		30.62 ± 2.41		33.96 ± 2.34	
Self-reported vaccination status						
Yes	397.19 ± 2.64	0.003	30.62 ± 2.38	0.005	34.12 ± 2.32	0.771
No	306.20 ± 2.67		24.55 ± 2.49		33.34 ± 2.45	

* for the level of significance of 0.05 according to Student *t* test for independent samples or One-Way ANOVA with Tuckey post hoc testing.

**Table 3:** Factors associated with measles, mumps, and rubella seronegativity.

Virus	Characteristics		Univariate			Multivariable				
			OR	95% CI OR	*p* *	OR	95% CI OR	*p* *	OR ^a^	*p* *^a^
Measles	Gender	Female	Ref.							
		Male	1.245	0.92–1.68	0.153					
	Age groups	19–20	1.060	0.71–1.58	0.776	1.054	0.70–1.58	0.800	1.071	0.748
		21–23	Ref.			Ref.			Ref.	
		24–26	1.087	0.81–1.46	0.582	1.138	0.84–1.54	0.398	1.157	0.361
		27–29	2.163	1.36–3.44	**0.001**	2.151	1.34–3.45	**0.001**	2.155	**0.002**
	Region of origin	Major urban	Ref.							
		Other urban or rural	1.065	0.83–1.37	0.619					
	Nursery/kindergarten attendance	No	Ref.							
		Yes	1.047	0.71–1.55	0.818					
	Migration	Yes	Ref.							
		No	1.012	0.79–1.30	0.924					
	Self-reported vaccination status	Yes	Ref.			Ref.			Ref.	
		No	1.684	1.15–2.46	**0.007**	1.730	1.18–2.54	**0.005**	1.701	**0.007**
Mumps	Gender	Female	Ref.							
		Male	1.321	0.98–1.78	0.066					
	Age groups	19–20	Ref.			Ref.			Ref.	
		21–23	1.522	0.99–2.35	0.057	1.525	0.99–2.36	0.058	1.496	0.053
		24–26	1.734	1.10–2.74	**0.019**	1.796	1.13–2.85	**0.013**	1.771	**0.009**
		27–29	2.304	1.27–4.18	**0.006**	2.217	1.21–4.05	**0.010**	2.179	**0.006**
	Region of origin	Major urban	Ref.							
		Other urban or rural	0.980	0.77–1.26	0.875					
	Nursery/kindergarten attendance	No	Ref.							
		Yes	1.483	0.98–2.25	0.063					
	Migration	Yes	Ref.							
	Self-reported vaccination status	No	1.045	0.81–1.34	0.731					
		Yes	Ref.			Ref.			Ref.	
		No	1.555	1.07–2.27	**0.022**	1.675	1.14–2.46	**0.009**	1.646	**0.011**
Rubella	Gender	Male	Ref.							
		Female	1.247	0.62–2.50	0.534					
	Age groups	19–20	Ref.							
		21–23	3.073	0.94–10.09	0.064					
		24–26	2.135	0.61–7.54	0.239					
		27–29	1.880	0.37–9.52	0.446					
	Region of origin	Other urban or rural	Ref.							
		Major urban	1.408	0.83–2.40	0.207					
	Nursery/kindergarten attendance	No	Ref.							
		Yes	2.535	0.78–8.20	0.121					
	Migration	Yes	Ref.							
		No	0.643	0.37–1.13	0.122					
	Self-reported vaccination status	Yes	Ref.							
		No	1.430	0.66–3.09	0.363					

* for the level of significance of 0.05, values that differ significantly are marked in bold, ^a^ Adjusted for gender, region of origin, attending nursery/kindergarten, migration.

## Data Availability

The data that support the findings of this study are available from the corresponding author upon reasonable request.

## Supplementary Materials

The following supporting information can be downloaded at: https://www.mdpi.com/article/10.3390/vaccines13070700/s1, Supplementary Table S1: Double and triple negativity according to age groups, Supplementary Table S2: Distribution of measles and mumps seronegativity among rubella negative/negative and equivocal individuals, Supplementary Table S3: Correlation between anti-measles, anti-mumps, and anti-rubella IgG antibodies.

## Abbreviations

The following abbreviations are used in this manuscript:

MMR	Morbilli-Mumps-Rubella
HCW	Healthcare workers
IgG	Immunoglobulin G
GMC	Geometric Mean Concentration
WHO	World Health Organisation
CRS	Congenital Rubella Syndrome
SAGE	Strategic Advisory Group of Experts
EU/EEA	European Union and European Economic Area
MM	Morbilli-Mumps
ECDC	European Centre for Disease Prevention and Control
AP	Autonomous Province
ELISA	Enzyme linked immunosorbent assay
