# Supplementary material for: Seroprevalence of Measles-, Mumps-, and Rubella-Specific Antibodies in Future Healthcare Workers in Serbia: A Cross-Sectional Study
Source: Vaccines (Basel). 2025 Jun 27;13(7):700. doi: 10.3390/vaccines13070700 (PMC12298484; doi:10.3390/vaccines13070700)
Supplement: Supplementary file 1 [file vaccines-13-00700-s001.zip › Supplementary Files.pdf]

Supplementary Table S1. Double and triple negativity according to age groups

Generation/Age group	Double negativity (anti-measles and anti-rubella)		p*	Triple negativity (anti-measles, anti-mumps, and anti-rubella)		p*
	Yes	No		Yes	No	
1995-1999	7 (2.5)	273 (97.5)	0.603	5 (1.8)	275 (92.2)	0.435
2000-2005	31 (3.1)	970 (96.9)		26 (2.6)	975 (97.4)	
19-20	2 (1.3)	157 (98.7)	0.447	1 (0.6)	158 (99.4)	0.395
21-23	24 (3.5)	657 (96.5)		20 (2.9)	661 (97.1)	
24-26	9 (2.5)	346 (97.5)		8 (2.3)	347 (97.7)	
27-29	3 (3.5)	83 (96.5)		2 (2.3)	84 (97.7)	

*for the level of significance of 0.05 according to chi-square test

Supplementary Table S2. Distribution of measles and mumps seronegativity among rubella negative/negative and equivocal individuals

Rubella	Measles negative, n (% of rubella, % of all)	Mumps negative, n (% of rubella, % of all)
Negative, n=58	45 (77.6, 3.5)	38 (65.5, 2.9)
Negative + equivocal, n=102	72 (70.6, 5.5)	63 (61.8, 4.9)

Supplementary Table S3. Correlation between anti-measles, anti-mumps, and anti-rubella IgG antibodies (Pearson coefficient of linear correlation r and p value are reported)

1995-2005	Anti-measles	Anti-mumps	Anti-rubella
Anti-measles	1	r = 0.320, p < 0.001	r = 0.314, p < 0.001
Anti-mumps		1	r = 0.402, p < 0.001
Anti-rubella			1
1995-1999			
Anti-measles	1	r = 0.347, p < 0.001	r = 0.314, p < 0.001
Anti-mumps		1	r = 0.336, p < 0.001
Anti-rubella			1
2000-2005			
Anti-measles	1	r = 0.318, p < 0.001	r = 0.320, p < 0.001
Anti-mumps		1	r = 0.428, p < 0.001
Anti-rubella			1
